# Where Technology Leads, the Problems Follow. Technosolutionism and the Dutch Contact Tracing App

**DOI:** 10.1007/s13347-024-00807-y

**Published:** 2024-10-28

**Authors:** Lotje E. Siffels, Tamar Sharon

**Affiliations:** Interdisciplinary Research Hub On Digitalization and Society (iHub), Faculty of Philosophy, Theology and Religious Studies, Department of Philosophical Ethics and Political Philosophy, Radboud University, Erasmusplein 1, 6525 HT Nijmegen, The Netherlands

**Keywords:** Technosolutionism, Big Tech, Contact tracing apps, COVID-19, Constructivism, Sphere Transgressions

## Abstract

In April 2020, in the midst of its first pandemic lockdown, the Dutch government announced plans to develop a contact tracing app to help contain the spread of the coronavirus – the *Coronamelder.* Originally intended to address the problem of the overburdening of manual contract tracers, by the time the app was released six months later, the problem it sought to solve had drastically changed, without the solution undergoing any modification, making it a prime example of technosolutionism. While numerous critics have mobilised the concept of technosolutionism, the questions of how technosolutionism works in practice and which specific harms it can provoke have been understudied. In this paper we advance a thick conception of technosolutionism which, drawing on Evgeny Morozov, distinguishes it from the notion of technological fix, and, drawing on constructivism, emphasizes its constructivist dimension. Using this concept, we closely follow the problem that the Coronamelder aimed to solve and how it shifted over time to fit the Coronamelder solution, rather than the other way around. We argue that, although problems are always constructed, technosolutionist problems are *badly* constructed, insofar as the careful and cautious deliberation which should accompany problem construction in public policy is absent in the case of technosolutionism. This can lead to three harms: a subversion of democratic decision-making; the presence of powerful new actors in the public policy context – here Big Tech; and the creation of “orphan problems”, whereby the initial problems that triggered the need to develop a (techno)solution are left behind. We question whether the most popular form of technology ethics today, which focuses predominantly on the *design* of technology, is well-equipped to address these technosolutionist harms, insofar as such a focus may preclude critical thinking about whether or not technology should be the solution in the first place.

## Introduction

‘If the app is the answer, then what is the actual question?’

Pieter van Boheemen^1^

At the outbreak of the COVID-19 pandemic in early 2020, governments and health authorities around the world turned to digital technology for support in addressing the rapid spread of the virus. Particularly promising was the idea of automating contact tracing; a public health strategy for containing infectious disease outbreaks that involves identifying infected individuals and informing the people they have been in contact with that they are at risk (EC, 2020; WHO, 2020). Digitizing (part of) the contact tracing work would enable the employment of this tried-and-tested method on the large scale of infections that COVID-19 brought with it (de Jonge, 2020; Ferretti et al., 2020; Wacksman, 2021). At the same time, digital contact tracing raised substantial privacy and surveillance risks (Ada Lovelace Institute, 2020; McGee et al., 2020), had a high probability of false positives (Lee, 2020; Vaughan, 2020), and its overall effectiveness was (and remains) questionable (Chiusi, 2021; ter Haar et al., 2023). Many countries ended up developing smartphone-based apps that automatically trace points of contact between individuals, and alert users when they have been in contact with someone carrying the virus. The design of the apps and its consequences for citizens’ privacy was fiercely debated and differed per country, with the Bluetooth-based and decentralized design being the most widespread (Shahroz et al., 2021; Lanzing et al., 2022). At the time of writing, most of the apps are no longer in use (EC, n.d.).

On April 7 2020, during the first lockdown, the Dutch government also announced plans to develop a contact tracing app, the *Coronamelder*. The announcement was a response to the overburdening of manual contact tracers, who could not keep up with the growing need for contact tracing. Half a year later, in the fall of 2020, the Coronamelder was released for public use. But where initially the app was supposed to address the overburdening of manual contact tracers by providing support, the app now no longer seemed to have anything to do with this overburdening. The aim of the app had changed drastically. In this paper we contend that throughout the process of developing and rolling out the Coronamelder, the problem the app was initially meant to solve changed over time without the solution – a smartphone-based contact tracing app – undergoing any modification. We argue that this makes it a prime example of technosolutionism.

Numerous critics have argued that the pandemic has shown how, in times of crisis, there is a tendency to turn to digital solutions to address public policy problems (Klein, 2020a, 2020b; Morozov, 2020). Many references to technosolutionism in relation to contact tracing apps were made, both in general (Harjric, 2020; Michael et al., 2020; Mann et al., 2022), and in the Dutch context (Appelman et al., 2020). However, with some exceptions (Campbell-Verduyn & Gstrein, 2024; Maschewski & Nosthoff, 2022), there has been little effort to describe the political development and rollout of contract tracing apps and the specific role technosolutionism played in it. Moreover, most discussions of contact tracing apps and technosolutionism make use of what we call a *thin* conception of technosolutionism. This overlooks its constructivist dimension and equates it with the simpler concept of “technological fix”, thereby failing to recognize the potency – and dangers – of technosolutionist thinking in public policy contexts, and how it might be prevented in the future.

Through this article, we hope to contribute to the literature on technosolutionism in several ways: by elaborating a thick conception of technosolutionism that embraces a constructivist approach, by showing what technosolutionism in the context of public policy making does, and by showing which harms a technosolutionist problem construction can provoke. We do this by following the problem that the Coronamelder was supposed to solve, and how it shifted to fit the Coronamelder solution, rather than the other way around, in the period between April and December 2020. Our analysis focusses specifically on the way government officials who were involved in the development of the app conceptualized the problem it sought to solve. We reviewed policy documents during this time that highlight the purpose and development of the app, including advice from committees, press conferences, parliamentary debates and letters from the Minister of Health to inform Parliament. We specifically sought out policy documents where the aim or purpose of the app are described, explicitly and implicitly, as well as the app’s effectiveness. Subsequently, to clarify our findings we had several conversations with persons involved in the development of the app, amongst whom government officials.

The paper proceeds as follows. In section 2, we distinguish between a *thick* and *thin* conception of solutionism, where the thick conception follows Morozov’s (2013) definition of technosolutionism, which involves a constructivist and temporal element of looking back from the solution and constructing a problem that justifies its implementation. Taking inspiration from Latour (2003), furthermore, we argue that it is not the fact that problems are constructed per se that is problematic with technosolutionism. Rather, that technosolutionist problems are not constructed *well*. In the context of public policy making, we contend, good problem construction implies careful deliberation and discussion of alternative solutions. Something we find to be painfully lacking in technosolutionism, we argue in Section 3, as we show how the problem definition follows the solution and is twisted to fit the solution, rather than the other way around. In Section 4 we zoom in on how technosolutionist problems are constructed. We show several moments of contestation, negotiation and stabilization in the construction of the problem that the Coronamelder sought to solve, which help us identify technosolutionism as a mechanism. In Section 5, drawing on our analysis, we describe some of the general harms of technosolutionism in the public policy context. These include a subversion of democratic decision making, the presence of new and powerful actors in the form of Big Tech companies, and the creation of what we call “orphan problems”: problems that initially triggered the development of a technosolution, but were abandoned when they no longer fit the technosolution. We believe that the ethical reflection which had been applied to the Coronamelder at the time was ill-equipped to address these harms that technosolutionism brings forth, since its focus was exclusively on the design of the technology. This focus precludes critical thinking about whether or not the technology should be developed in the first place. We contend that this type of ethical reflection is prevalent in ethics of technology and argue for a broader ethical reflection, one that includes consideration of democratic values and the question of how technological (vs. non-technological solutions) may impact them.

## Towards a Thick Conception of Technosolutionism

In the past decade, the term (techno)solutionism has become widespread among technology critics. “Solutionism” as a concept was coined by Dobbins (2009) in the context of city design and planning. It was first used to describe the ideology surrounding technologies and popularized as “technological solutionism” by Evgeny Morozov (2013) in his widely acclaimed critique of smart technology and the Silicon Valley mindset. It has proven to be a potent concept for calling out the promotion of simple technological solutions to complex societal problems. Morozov was not the first technology critic to identify this dynamic. In 1965, Alvin Weinberg famously coined the term “technological fix” to describe the use of technology to respond to problems that are typically defined as political, legal, organizational, cultural or social. Although Weinberg argued for the value of such technological fixes (Johnston, 2018), the term has prevailed as a pejorative one (Rosner, 2013), used to criticize the tendency to believe that “wicked problems” can have quick and easy technological fixes (Rittel & Webber, 1973).

Thinking of technosolutionism as a mindset, or even an ideology, which tries to fix all social problems with technology, generates interesting technology critiques. Morozov’s understanding of technological solutionism, however, differs from the notion of technological fix in important ways. ‘Solutionism,’ Morozov writes,

is not just a fancy way of saying that for someone with a hammer, everything looks like a nail; it’s not just another riff on the inapplicability of “technological fixes” to “wicked problems”. It’s not only that many problems are not suited to the quick-and-easy solutionist tool kit. It’s also that what many solutionists presume to be “problems” in need of solving are not problems at all. (2013, 6)

In other words, solutionism is not just about misunderstanding complex societal problems as ones that technology can address, it is about constructing problems as such: about re-interpreting phenomena, which may not be problematic at all, *qua* problems, which technology would then be best placed to solve. Solutionism implies a *backward*-looking orientation – a reformulation or recasting of an existing phenomenon as a problem – rather than a *forward*-looking orientation that decision-making (ideally if not in practice) entails, moving from a problem towards a solution. Thus, Morozov argues, sleep, becomes a “problem” once sensors which can collect data about light, air-quality, sound and temperature can be used to monitor sleep cycles in one’s bedroom (2013, 227). Once such solutions exist, sleep can be recast as a problem to optimize. The concept of technosolutionism thus implies paying close attention to how problems are constructed or transformed by solutionism. ‘In solving the “problem”, solutionists twist it in such an ugly and unfamiliar way that, by the time it is “solved”, the problem becomes something else entirely’ (2013, 8).

Morozov points out that the problem and its construction should be a main focus in any critique of technosolutionism. This insight has much to contribute to discussions on the relationship between technological solutions and societal problems, arguably more than even Morozov initially intended by appealing to the notion of construction. Indeed, from a constructivist perspective, as developed most notably in the philosophy of science (Hacking, 1999) and Science and Technology Studies (Latour & Woolgar, 1979), problems – just as mental illness labels or scientific facts – are always constructed. Such things are not just given, out there to be discovered. They are made, through negotiation, contestation, deliberation and other power struggles, and much work goes into making them.^2^ For theorists such as Latour, this means that we should focus on constructing something like scientific facts *well.* Constructivism should, according to Latour, be seen as an ‘appeal for the extension of *care and caution*, a request to raise again the question: “How can it be built *better*?”’ (Latour, 2003, p.42, original italics). A distinction thus emerges between *badly*-constructed and *well*-constructed problems, which is not part of Morozov’s original definition of technsolutionism but which we believe, as will be discussed, contributes much to this discussion. Namely, it allows one to focus on how the problem definition for which a technosolution is proposed came to be and to question if this was done well.

This constructivist dimension, both Morozov’s variant and the more radical one, is missing in the notion of technological fix and makes technosolutionism a much richer concept. Still, and despite Morozov’s attempt to clearly distinguish between both concepts, in the literature technosolutionism is frequently reduced (back) into the thinner notion of technological fix (see for example Green, 2020; Hajric, 2020; Michael et al., 2020; Ferrari, 2020; Maréchal, 2021; Maschewski & Nosthoff, 2022; Mann et al., 2022; among many others).^3^ By referring to the idea that there is a ‘techno-entrepreneurial solution to every social problem’ or ‘a technological hammer for every social nail’ (Nachtwey & Seidl, 2020), many authors, while mobilizing technosolutionism in name, are actually referring to the technological fix. Even scholars who have recently used related concepts to describe a similar phenomenon tend to fall back onto the thin concept of technological fix. Thus, Broussard (2018) speaks of “technochauvinism”: ‘the belief that tech is always the solution’, Green (2020) speaks of “tech goggles”: ‘when we conceive every issue as a technology problem’, and McDonald (2020) speaks of a “technology theatre”: ‘the practice of focusing public attention on elaborate, ineffective procedures to mask the absence of a solution to a complex problem’.

This is regrettable, as the thick conception of technosolutionism offers a more valuable analytical lens through which to scrutinize the use of digital tools in the arena of public policy, especially when it implies the stronger variant of constructivism. Rather than focusing (merely) on the question of the (in)adequacy of the proposed technological solution, it allows us to see more clearly the power that these technologies and the discourses surrounding them hold, by indicating how problems are constructed and re-constructed to accommodate these technologies. This, in turn, allows us to better critically examine the space taken up by, and the resources spent on, these technologies. The thick conception also offers more methodological guidance for recognizing technosolutionism. It invites us to follow the problem and how it changes through time. Finally, it opens up the question of whether this problem has been constructed *well*, if other problem definitions did or could have existed and what has been left out by defining the problem in a way that can be solved by a technology. Indeed, as we will argue, a careless construction of problems can undermine support for more ambitious, progressive and comprehensive policies which technologies-qua-solutions cannot address. In the next section, we show how applying this thick conception of technosolutionism is useful for analyzing the decision-making process around the adoption of the Dutch contact tracing app.

## The Many Problems of the Coronamelder Solution

When the government first announced that it would develop a contact tracing app, in early April 2020, the envisioned purpose of the app was that it would solve a specific problem. At that time, the Netherlands was in lockdown to prevent further spread of the coronavirus. As in other countries, manual contact tracing work, normally undertaken by the Dutch Municipal Health Services (*Gemeentelijke Gezondheidsdienst* or *GGD*),^4^ was at this time the most important virus containment strategy. But the GGDs across the country were overwhelmed. They did not have the human capacity to manage the contact tracing needs required by the rapidly spreading virus, let alone to manage this when lockdown measures would be lifted, as the government wished to do swiftly. This lack of capacity of the GGDs was seen as one of the main obstacles to lifting lockdown measures^i^ 5 (de Jonge, 2020). An app which supported or replaced some of the work undertaken by the GGD by automating contact tracing would, it was hoped, solve this problem of undercapacity. While this aim was clear in all policy documents discussing the need for a contact tracing app in April 2020, at the launch of the Coronamelder several months later, undercapacity is no longer mentioned in relation to the app’s development, and its purpose is described in very different terms. Even more so, once launched, the Coronamelder had nothing to do with supporting the work that the GGD carries out. In this section we trace how the problem the Coronamelder was supposed to solve came to be reconstructed as a problem that it actually addressed. These shifts are summarized in Table 1.

**Table 1:** Phases in the construction of the technosolutionist problem of the Coronamelder solution

Phase	I (early april 2020)	II (late april 2020)	IIIA (may - october 2020)	IIIB (october - december 2020)
Main Events	• Outbreak COVID-19 in NL • Announcement development app	• Appathon	• Release Google/Apple API • Publication of OoR^a^	• Release Coronamelder
Solution Definition	A smartphone-based contact tracing app	A smartphone-based contact tracing app	A smartphone-based contact tracing app	A smartphone-based contact tracing app
Status of solution	App is a vague idea, some apps are being developed in other countries	Proposals for prototype apps to be tested during the appathon	Coronamelder developed based on the API released by Google and Apple	Coronamelder released
Problem definition	• GGD is overburdened and needs support	• GGD is overburdened and needs support • Manual contact tracing cannot find all relevant points of contact • Manual contact tracing cannot alert possibly infected people fast enough • There is not enough insight into how the virus spreads	• Manual contact tracing is too slow and cannot find all relevant contacts	• Manual contact tracing is too slow and cannot find all relevant contacts
Key words	*Supporting*	*In addition to*	*Better and faster*	*Better and faster*
Criteria of effectiveness	‘We have to try to achieve that 60% [uptake]’^ii^	‘We just have to start working with it and then see what the effectiveness is’^iii^	‘We just have to start working with it and then see what the effectiveness is’	‘Intended effect: find infected people faster’^iv^

^a^Overview of Requirements for a digital solution to supplement source and contact research

**Table 2:** Overview of main organizations involved in the development of the Coronamelder

Organization	Function
GGD	The Municipal Health Services, (Gemeentelijke Gezondheidsdiensten) are 25 regional offices addressing issues of public health, among which virus containment, vaccination programs, air quality and population surveys. They are bundled in the umbrella organization *GGD GHOR*, which is the representative of public health and safety in the Netherlands
OMT	The Outbreak Management Team is an emergency team existing of representatives from the major public health institutions dealing with infectious diseases and virus containment. An OMT is set up when an infectious disease poses a large threat and always consists of these regular participants: the director of the *Centre for Infectious Disease Control,* the head of the *National Coordination of Infectious Disease Control* (*Landelijke Coördinatie Infectieziektebestrijding* or *LCI*) from the *National Institute for Public Health and Environment (Rijksinstituut voor Volksgezondheid en Milieu,* or *RIVM*), and a GGD doctor specialized in infectious diseases
Advisory Committee on Digital Support to Combat COVID-19^a^	A committee of experts in epidemiology, virology, technology, privacy and security who advised the Ministry of Health about digital support for fighting COVID-19 from 20–05-2020 until 22–02-2022
Code for NL	Special Slack channel for discussing the development of the Dutch contact tracing app. Set up by the *Waag Futurelab*, a Dutch civil society organization that facilitates critical reflection on the use of technology, and involves tech developers and designers, journalists, scholars and activists, as well as government employees and civil servants

^a^Begeleidingscommissie digitale ondersteuning bestrijding COVID-19

### From “Support” to “Add-on”

In early April 2020, when the first announcements concerning the development of an app were being made, one term was central to its narrative: *support*. Furthermore, it was clearly communicated that the app to come would seek to support the GGD, and the specific problem of undercapacity. Indeed, the development of the app was instigated by the advice that the Dutch *Outbreak Management Team* (OMT) – an emergency organ established to advise the government on virus containment – put forward, which urged to quickly investigate ‘possibilities to *support* contact tracing using mobile applications’^v^.^6^ Minister of Health Hugo de Jonge announced in a press conference that ‘we need those apps to *support* the normal GGD work’^vi^ (de Jonge, 2020). Subsequently, a call went out to companies to submit prototypes for ‘smart digital solutions’^vii^ (Ministerie VWS, 2020a), which repeated the advice to, ‘as fast as possible investigate the possibilities for *supporting* contact tracing using mobile applications in order to reduce the burden on the GGD’^viii^. In this call, applicants were encouraged to familiarize themselves as much as possible with analogue contact tracing – in order to best come up with a solution to support it. Similarly, *support* was the key word in the various parliamentary briefings and discussions about the app. De Jonge, for example, emphasized during one of the parliamentary debates in April 2020, that the right questions to ask about the forthcoming app were if it, ‘actually *supports* the normal work process of the GGD. (…) That is the purpose [of the app] and it must fulfill that purpose.’^ix^ Additionally, the Ministry’s Chief Information Officer (CIO) stated in late April that, ‘in everything that has been done, that question has always been at the forefront, namely the purpose of exploring the possibility of digital *support* [for manual contact tracing]’^x^. In short, during the month of April 2020, supporting the work of the GGD was the purported aim of the app, which would help solve the problem of the GGD’s overburdening and undercapacity.

One month later, the Ministry of Health released a document called the *Overview of Requirements for a Digital Solution in Addition to Contact Tracing* (Ministerie VWS, 2020c)*,* which would act as a guideline for the further development of the app*.* As the title suggests, from this moment on, the contact tracing app to come was no longer discussed in terms of supporting the work of the GGD, but in terms of being an *addition to*, and separate from, the work of the GGD. Correspondingly, in his letters to Parliament of this time, Minister de Jonge recurrently describes the need for an app, ‘*in addition to* existing contact tracing’^xi^, and of the app as ‘*add[ing] to* regular contact tracing’^xii^. This terminology was further taken up by the *Advisory Committee on Digital Support to Combat COVID-19*, a committee of experts in epidemiology, virology, technology, privacy and security who advised the Ministry of Health about digital support for fighting COVID-19. Their advice to the Minister speaks of: ‘The added value of the notification app *on top of* regular contact tracing’^xiii^. During the parliamentary debate of September 2, 2020, the Minister emphasized again that the app will not so much support the work of the GGD, but that it had value ‘*in addition to* contact tracing’^xiv^.

This shift in the description of the app’s purpose, from *support* to *add-on*, can also be identified, albeit more subtly, in discussions about what the app should *not* do – namely, interfere with, or pile on top of, the work of the GGD’s. Indeed, one of the points made in the *Overview of Requirements* states that ‘the realization and implementation [of the app] should not have any negative effect on the existing processes of the GGD’^xv^ (Ministerie VWS, 2020c). This concern was also voiced in a report presented in July 2020 by a panel of experts, who were tasked with providing an ethical analysis of the app, which was then still in development: ‘It is important to determine,’ the report states, ‘how much work the app will entail for the GGD: can the current system deal with this?’^xvi^ In other words: by this time, the app’s aim was no longer to solve the overburdening of the GGD, but to do something in addition to the GGD, which it is important to make sure does not in the end increase the workload of the GGD.

The reformulation of the app’s aim from providing support to being an add-on is not just a semantic difference but reflects a substantive and consequential shift in how the problem was being defined. During the course of one month, the purpose of the app had gone from supporting the work done by the GGD to not getting in the way of what the GGD does while doing the same thing. In the process, the GGD’s stakes in the development of the app shifted from being its main beneficiary to becoming a bystander at risk of being negatively affected by the implementation of the app. In other words, there were now concerns that, rather than alleviate the overburdening of the GGD, which had originally been identified as the problem that needed solving, the app could actually aggravate this problem. This shift in the purpose of the app and the problem it sought to solve took one more turn when the actual Coronamelder was released, and its aim was again redefined – not as supporting or even simply adding to the work of the GGD, but as *improving* upon the work of the GGD.

### Better and Faster

On April 18 and 19 2020, the Ministry of Health organized an *appathon*; an event where the prototypes for a contact tracing app sent in by several commercial parties would be tested by a group of experts in an open and transparent process. A close look at the statements and the discussions taking place during the appathon, shows that the original problem that the app purported to solve – the overburdening of the GGDs – was not abandoned all at once. One way this shift came about was when it became clear, during the appathon, that an app could provide other benefits too. In other words, that the proposed solution may be more attuned to a different problem.

The appathon, attended by representatives of the companies whose prototypes were selected, the CIO of the Ministry of Health, the Minister himself, a group of experts invited to test the prototypes, and the director of the GGD, opened with the following statement by a representative of the Ministry: ‘We are gathered this Saturday to solve one of the big questions of our time: how can we better map the spread of the coronavirus? How can we trace the contacts of infected people better and faster?’ (Ministerie VWS, 2020b)^xvii^ As the appathon proceeded, these two aims, which we summarize as contact tracing that is *better* and *faster* relative to the GGD’s existing work, became increasingly dominant. When the moderator of the appathon asks the director of the GGD, ‘What can the app do that you can’t?’^xviii^, the director responds by acknowledging both aims: that an app can be faster than the GGD because it can send out immediate notifications (*faster)*, whereas a manual contact tracer would need to investigate, in conversation with an infected person, who might have been in their proximity and find out how to reach them. On top of that, an app could also, he explains, contact people whom an infected person does not know they were in contact with (*better)*, such as a fellow diner in a restaurant, which would be particularly useful in a post-lockdown phase. The CIO of the Ministry also affirms the notions of *better* and *faster* contact tracing by saying: ‘you wouldn’t have to rely on people’s memory, and you can trace contacts that we may not be aware of or have forgotten, thus speeding up the process’^xix^.^7^

In a month’s time, these additional benefits come to be defined as the main aims of the app. In the *Overview of Requirements*, in letters from the Minister to Parliament and in the report made by the Advisory Committee, it is the added value of the app in terms of faster detection and notification of contacts, including anonymous contacts, which are then deemed to be the main purpose of the app. In other words, what started off as additional benefits of the app came to be used to redefine the problem that the app would solve: that the contact tracing work of the GGD is too slow and not thorough enough. Before the app came along, these were not problems that needed solving. The additional benefits and newly constructed problems then became *the* problem the app was meant to solve.

### Criteria of Effectiveness that Emerge Along the Way

An additional way to identify these shifts in problem definition is to trace how the criteria of effectiveness of the solution evolved in tandem with the problem definition (see Table 3). Initially, when the problem the app was meant to solve was defined as the overburdening and undercapacity of the GGD, the effectiveness of the app was discussed in terms of how it supported the GGD. For example, early on it was said that if the app would have an important uptake (at least 60%), it could replace some of the manual work of the GGD.^xx^ Later on, this criterion was let go as the problem definition changed from *supporting* the GGD to being an *add-on* to the GGD’s work. In a parliamentary debate on September 3 2020 the Minister said: ‘Of course, you want the app to be used as much as possible, but I’m not going to put a number on that beforehand. (…) I think we just have to start working with it and then see what the effectiveness is.’^xxi^

It was not until November 2020 that any criteria of effectiveness were disclosed. In an update to Parliament, the Minister offered an evaluation of the app. Suddenly, effectiveness was not evaluated in terms of uptake – only 22,5% of the Dutch population had downloaded the app, which is a far cry from the 60% threshold, albeit still higher than in most European countries^8^ – but in terms of the *better* and *faster* criteria. Effectiveness was evaluated by how many people received a notification from the app who had not (yet) received a call from the manual contact tracers.^9^ The number was 99,8% and was immediately adopted as a proxy for the app working *faster* than the manual contact tracers.^xxii^ The criteria of effectiveness thus, rather than being predefined, shifted along with the problem definition to become criteria the app could meet.

**Table 3:** Changes in problem definitions and criteria of effectiveness throughout the phases

Phase	Key words	Problem definition	Criteria of effectiveness
I (early april 2020)	*Support*	• GGD is overburdened and needs support	‘We have to try to achieve that 60% [uptake]’^xxiii^
II (late april 2020)	*In addition to*	• GGD is overburdened and needs support • Manual contact tracing cannot find all relevant points of contact • Manual contact tracing cannot alert possibly infected people fast enough • There is not enough insight into how the virus spreads	‘We just have to start working with it and then see what the effectiveness is’^xxiv^
IIIA (may-october 2020)	*Better and faster*	• Manual contact tracing is too slow and cannot find all relevant contacts	‘We just have to start working with it and then see what the effectiveness is’
IIIB (october-december 2020)	*Better and faster*	• Manual contact tracing is too slow and cannot find all relevant contacts	‘Intended effect: find infected people faster’^xxv^

## The Construction of Technosolutionist Problems: Contestation, Negotiation and Stabilization

It is not easy to catch technosolutionism in the act. Problem definitions, and their accompanying aims, criteria of evaluation and effectiveness, may shift abruptly, subtly or incrementally, in ways that require careful attention. One must follow the problem closely. In Section 3, we showed *that* the problem definition for which the Coronamelder was a solution shifted. In this section, inspired by constructivist approaches in the philosophy of science and Science and Technology Studies, we analyze what happened during those shifts in problem definition, i.e. how they came about.

### A Constructivist Approach to Technosolutionism

When doing constructivist work, it is important to specify exactly *what* is being constructed (Hacking, 1999). In our case, we are argue that problems, and more specifically problems for which government policy and spending should offer a solution, are not inevitable or given, but constructed. As mentioned earlier, constructivist scholars of science (e.g. Bloor, 1976; Collins, 1981; Latour & Woolgar, 1979) have studied at length the construction of scientific facts. In their seminal *Laboratory Life: The Construction of Scientific Facts* (1979), for example, Latour and Woolgar (1979) show that the construction of scientific facts is a process of contestation, negotiation and finally stabilization of statements, after which the resulting fact loses its constructed character, and after which the work that went into constructing it is forgotten. ‘Facts are constructed,’ they write, ‘in such a way that, once the controversy settles, they are taken for granted’ (1979, p. 183). But this “before” and “after” can be observed.

Of course, facts are not the same kind of things as problems. Still, we believe important parallels can be drawn between the work of construction that goes into making both. As with scientific facts, while it is difficult to pinpoint the exact point at which the problem the Coronamelder sought to solve was reconstructed, it is possible to ascertain a “before” and “after”, i.e. a “stabilization” process. And as with scientific facts, once a problem definition has been constructed which fits a given solution, it comes to be seen as having always been there, while the work that went into constructing it, including the pushing aside of the initial problem, is forgotten. Furthermore, as we show below, it is possible to identify moments of “contestation” and “negotiation” in the construction of our problem.^10^

### Contesting the Problem Definition

From the documents we analyzed for the Coronamelder case, we identified what can be seen as two moments of contestation regarding the problem definition that the Coronamelder was presented as solving. Importantly, these are not so much moments when contestation *about* the problem definition was taking place. That is, this contestation was not explicit deliberation about the problem definition. Rather, these are moments when different problem definitions clashed and where contestation about the *lack of* deliberation were voiced, and which signal a “before” and “after” with regards the stabilization of the problem definition.

The first moment we identified took place early on in the process. When the government announced that it would develop a contact tracing app, 61 scholars, scientists and experts wrote an open letter, on April 13 2020. They were concerned about technosolutionism; that the implementation of an app would become the main focus in the government’s COVID-19 policy. They warned that: ‘Digital technology may contribute to solving a social problem but is rarely *the* solution to a social problem.’ The authors urged the government to make clear which problems it sought to address and to critically assess whether an app would solve them. The letter also emphasized several times that the possibility of not using an app should never be excluded (Muller et al., 2020). As our analysis above shows, the government did not share these concerns.

A second moment of contestation can be identified in an exchange that took place between the Minister of Health and the Advisory Committee. Similar to the authors of the open letter of April 13 2020, the committee was critical of the government’s focus on an app as a solution, as well as the lack of definitions of requirements and criteria of effectiveness. In a report published on May 28 2020 responding to the government’s *Overview of Requirements* [*OoR*] document which would act as a guideline for the development of the app, the committee stated:

The *OoR* seems to be working towards a specific solution, namely the creation of a specific notification app, instead of providing a concrete and generic overview of the preconditions and formulating requirements for this form of digital support.^xxvi^

Contestation, albeit somewhat concealed, is also conveyed here by the mismatch between how this report formulates the problem definition and how the *OoR* does. While the committee here is still referring to the app in terms of its aim to *support* the GGD’s work, the *OoR*, as we have shown in Section 3, was already discussing the app’s aim in terms of an *add-on* to the GGD’s work. Furthermore, the Minister’s response to the committee’s accusation of technosolutionism was to contest it. Not by engaging with it, but by ignoring it altogether and instead stating a newer problem definition the app sought to solve – namely that manual contact tracing is not accurate or fast enough – as if this had always been the problem definition. The aim of the app is now presented as a tool that will be an *add-on* to the work of the GGD rather than *support* it, because it is better and faster. This response by the Minister was conveyed in a letter to Parliament updating the state of affairs concerning COVID-19 on June 24, 2020:

The Advisory Committee recommends further specifying the purpose of the notification app and drawing up criteria of success and failure. The purpose [of the app] is very important to me. It is to trace those contacts that infected persons don’t remember, and quickly let them know they’ve been in contact with someone who is infected with the virus. This adds to the regular contact tracing work and leads to more, and faster, alerted contacts.^xxvii^

This exchange shows, again, how the aim of the app shifted, but also that there was some contestation around this, albeit contestation which never made it to the level of democratic deliberation. Somewhat surprisingly, the Advisory Committee, at first critical of the solution-oriented focus of the *OoR*, espoused the Minister’s new definition problem shortly after, despite its criticisms never having been properly addressed. On July 3 2020, the Committee published a new report which adopts without question or contestation the aim of the app as the Minister described it. By July 2020, any voiced contestation was settled. The shift from one aim and problem definition to another was a means of dismissing critique and settling any contestation; “stabilization” in Latour and Woolgar’s terminology, had been achieved.

### Contestation on the Sidelines

Most of our analysis has focused on publicly available policy documents, through which we have traced a shift in aims and problem definitions. Contestation and negotiation concerning these definitions can also be gleaned from another important source, the Slack channel *Code for NL*,^11^ where abundant discussions around the Coronamelder took place during this time. This channel was set up by the *Waag Futurelab*, a Dutch civil society organization that facilitates critical reflection on the use of technology, and was visited by tech developers and designers, journalists, scholars and activists, as well as government employees and civil servants. Contestation was quite rife on the platform during the first two months of the app’s development. For example, on April 19 2020, while the appathon was in full speed, a conversation between two users pitted different problem definitions that co-existed against each other:

User 1: I don’t think finding contacts between people who know each other will be the difficulty for the GGD. I know very well who I visit (…). Right now, it’s about that stranger on the train, who the GGD can’t reach.

User 2: But that isn’t the argument the GGD upheld as to why they want an app, is it? Rather, they’ve said it’s a scalability issue. Not a problem of too little data.’^xxix^

In this exchange, there is contestation over the different problem definitions, and a recognition that there has been a shift in the problem definition being presented at the appathon. We saw many similar exchanges in the Slack channel during this period, where discrepancies are recognized between a “before” and “after”, and where motivations about shifts in problem definitions are often commented on with cynicism.^12^ As in the policy discussions, this contestation dies out in the months that follow, when the new problem definition stabilizes.

### Negotiation: The Co-existence of a Plurality of Problem Definitions and Technology Tweaks

In our analysis, we identified two forms of negotiation of the problem definition, which preceded stabilization of the technosolutionist problem. The first is a broadening of the problem definition into several co-existing problems, before these were narrowed back down to one problem definition. The second is changes – or tweaks – in the technology, which the problem definition had to follow. Here, the definition of the problem is redefined to fit the affordances^13^ of the technology. In both forms of negotiation, the developments and adjustments to the solution triggered and determined changes in the problem definition, rather the than other way around.

We distinguished three main phases in the construction of the problem (see Table 4). In the first phase, in early April 2020, there was only one problem definition: an overburdening and undercapacity of the GGD, which needs support. While the solution was already there – a smartphone-based contact tracing app – this technology was still quite vague, and based on apps that were being developed in other countries. Phase II began with the appathon on April 18 2020. The appathon was an important site of negotiation, because it was here that different versions of the given solution – or prototypes – could be tested. A plurality of problems came into being during this phase, as promises of what the app, still vague, could do. An app might be able to do more than just tackle undercapacity, it may also be able to find more contacts. Or an app may help us gain new insight into the spread of the virus. This second phase was characterized by the co-existence of numerous problem definitions that the app could potentially solve.

**Table 4:** The problem definition follows the tweaks in the technology

Phase	Tweaks in technology	Problem definition
I (early april 2020)	App is a vague idea, some apps are being developed in other countries	• GGD is overburdened and needs support
II (late april 2020)	Proposals for prototype apps to be tested during the appathon	• GGD is overburdened and needs support • Manual contact tracing cannot find all relevant points of contact • Manual contact tracing cannot alert possibly infected people fast enough • There is not enough insight into how the virus spreads
IIIA (may-october 2020)	Gapple API released	• Manual contact tracing is too slow and cannot find all relevant contacts
IIIB (october-december 202)	App finalized	• Manual contact tracing is too slow and cannot find all relevant contacts

Out of this plurality, some problem definitions were selected, and others were abandoned as the app became more concrete through a series of tweaks in the design process. The first of the tweaks was a consequence of the release of the Google and Apple (Gapple) API, and would initiate the third phase (IIIA). In April 2020, as many countries debated the benefits and risks of digital contact tracing apps as a pandemic management strategy, Apple and Google launched an API for contact tracing on which such apps could run. In light of the substantial privacy concerns that digital contact tracing raised, Apple and Google’s API involved specific privacy enhancing technical criteria which apps would need to conform with, namely Bluetooth and decentralized storage (Apple, n.d.). But decentralized storage was an ill fit with some of the problem definitions from phase II. Only centralized storage could actually provide insight into the spread of the virus and predict where clusters of infection might arise. Hence, if the Coronamelder app would run on this API – as neighboring countries were opting for – the aim of gaining insight into the spread of the virus would need to be abandoned. As will be argued in the next section, by deciding on these tweaks in the technosolution, Google and Apple were granted important decision-making power. For the same reason, such an app would not be able to address the initial problem, to help or replace the manual contact tracing work of the GGD, which requires the sharing of personal data, which the Gapple API could not facilitate. However, what such an app *could* do is find more points of contact than manual contact tracing could. Therefore, building the app on the Gapple API eliminated some functions and therewith affordances of the app: or which problems the app could solve.

Another tweak that influenced the construction of the problem was the finalization of the Coronamelder itself. As discussed, as the design of the app was finalized, it became clear that the speed with which it could identify contacts was a novelty in comparison to the work of the GGD, which in turn became an important part of the problem definition. The problem that was constructed now that the Coronamelder was finished, was that the contact tracing of the GGD was too slow and imprecise.

Finally, as we saw in Section 3, when the app was introduced and began to be used, in what we called phase IIIB, the criteria of effectiveness, and thereby the problem definition, was again slightly reformulated. As the results of the app in practice showed its affordances even more clearly, the problem could be constructed more precisely to fit what the app demonstrated it could do: find additional infected persons and warn them *faster* than they would have been warned by the GGD.

Even though it was already clear what the technology/solution would look like in broad strokes in the early phases while the technology was still abstract, the discrepancy between the problem definition and the solution became more apparent as the solution became more concrete, leading to shifts in the problem definition. With technosolutionism, as the solution becomes more concrete, its affordances become more apparent, and the construction of the technosolutionist problem follows these affordances.

## Technosolutionist Harms

Constructivism always carries a risk of relativism (Hacking, 1999, p. 3–5; Latour, 2013, p. 8 & 156). If there are no “real” problems and “fake” problems, how do we decide on which problems to focus? And what role can critique play? It may seem as if arguing that problems are constructed is the same as an invitation to regard all problems as equally worth solving. Here too, we take inspiration from Latour (2003), who explains that the notion of constructivism is promising *because* it shifts our attention away from merely describing facts as a given towards the work that is done in their construction, and distinguishing between well – and badly – constructed facts. Constructivism, Latour argues, is all too often seen as an invitation for *deconstruction,* but its aim is the opposite: ‘How long will it be before the word [construction] is heard not as a war cry to take up arms or hammers, but as an appeal for the extension of *care and caution*, a request to raise again the question: “How can it be built *better*?”’ (Latour, 2003, p.42, original italics).^14^ Similarly, we argue that problem definitions need to be constructed *well*, and that, especially when problem construction is taking place in the context of public policy, this has mostly to do with careful and rigorous deliberation. Our analysis shows that when technosolutionism is at work in the public policy setting, problem construction is not guided by careful and rigorous deliberation reflecting societal needs, but by the affordances of the technosolution. Technosolutionist problems, that are constructed so that a technological solution can address them, are hence badly constructed.

The previous sections aimed to aid in finding ways to catch technosolutionism in the act. But why is it important to be aware of the workings of technosolutionism? Why do we need to spot it? In this section, drawing on our case, we discuss three general harms that can result from technosolutionism in public policy settings. These are: a subversion of democratic decision-making processes; the involvement of tech corporations in public policy, or “sphere transgressions”; and the creation of what we call “orphan problems”, or the forsaking of initial or carefully constructed problems for problems that technosolutions can solve. We conclude with an additional point: none of the harms we list here can be adequately addressed by the most popular forms of technology ethics today, design ethics or value sensitive design. This is because, in terms of our analysis, they are *post-factum* – their intervention is already at the stage of solution design, rather than *ex-ante*, intervening at the stage where it is still possible to ask if technology should be proposed as the solution at all. This gives rise to an additional harm, whereby technology ethics today unwittingly plays into the hands of, and so strengthens, technosolutionist trends, and thereby may contribute to the harms of technosolutionism which we identify.

### Technosolutionism as a Subversion of (ideal) Democratic Decision-Making

In the context of public policy and deliberative democracy, the care and caution that is required to construct problems well involve an extensive deliberative process, which entails discussion of different problem definitions, debate about possible solutions that are weighed in terms of their effectiveness and proportionality, followed by the selection of a solution, which should then be monitored and evaluated. There is an implicit sequence here, from problem construction to solution selection, which technosolutionism flips on its head. Its backwards-looking orientation, from solution to problem, diverges significantly from how decision-making in public policy should proceed – at least ideally, if not in practice. The implication is that technosolutionism, as a form of decision-making, is at odds with deliberative democratic decision-making.

This depiction of technosolutionist vs. democratic decision-making, admittedly, requires some qualification. First of all, in practice, technosolutionism and deliberative democratic decision-making are not mutually exclusive. Both forms of decision-making may be present within a larger policy making process (which can make it more difficult to discern technosolutionism at work). In the case of the Coronamelder, the development of the solution – a smartphone-based contact tracing app – *did* follow the democratic decision-making process at first. The problem definition, pertaining to the overburdening and undercapacity of the GGD, was the result of deliberation, and included advice provided by the Outbreak Management Team. The democratic decision-making process, however, quickly veered into technosolutionism with the announcement of the development of an app to solve the problems of the GGD, which seemingly came out of the blue, rather than being the result of a rigorous parliamentary debate. Indeed, the announcement was a surprise to many, most notably to the directors of the GGD^15^ (Onzerzoeksraad voor Veiligheid, 2022, p. 128), and even to employees of the Ministry of Health (Martijn, 2023). After the sudden announcement of an app, this solution remained fixed. Though there was some discussion on the design and implementation of the solution, the solution itself was not the focus of those discussions.

The process could have then veered back to a democratic form of decision-making with the initiation of the appathon. An appathon can, in principle, act as the embodiment of a deliberative process, and indeed, hackathons often mobilize democratic values and ideals (Yuan & Gasco-Hernandez, 2019; Irani, 2015; Taylor & Clarke, 2018). The Coronamelder appathon was also intended to be an open, democratic process, where, in principle, anyone could answer the government’s call to contribute a prototype solution, and during which various prototypes would be tested and criticized.^16^ However, this democratic and deliberative framework was thwarted from the get-go by the way the appathon was set up. The call for participation, aimed at commercial technology developers, asked for prototypes which could be designed and tested in relation to an *already* determined solution – an app. In other words, the appathon was not set up as a space to deliberate if an app or a different (technological) solution would be the best fit to the given problem. Rather it was a space for testing different prototypes, or versions, of a predefined solution.^17^

Nor did the process regain a more democratic path when the appathon failed to bring forth a final prototype that could meet the demands of the government and other organizations.^18^ At this point the democratic decision-making would have prescribed a renewed deliberation beginning from the problem definition, to construct a solution definition which better fit the problem. Instead, the government stuck with the solution definition it had and redefined the problem which this solution could solve.

A second necessary qualification here is that we pit technosolutionism against an *ideal* of deliberative democratic decision-making, which does not do justice to the messy reality of decision-making in public policy, as a number of practice-oriented policy studies scholars would argue (see for example Lindblom (1959) and Cohen et al. (1972)).^19^ In practice, instead of a process whereby objectives are clarified from the start and then policies are chosen to achieve them – a model Lindblom calls the “rational-comprehensive model” – policy makers tend to select and define the means and ends of their policies simultaneously – what Lindblom calls the “method of successive limited comparisons” (Lindblom, 1959). However, while the “rational-comprehensive model” may indeed be an idealized model, the “method of successive limited comparisons” does not explain what we observed in the case of the Coronamelder either. We did not observe a co-constitution of problem and solution definitions in some reiterative process. Instead, we clearly observed a distinctive sequence from solution to problem definition, which conflicts with both models, and conflicts most starkly with the idealized model of democratic decision-making. Observing technosolutionism at work in our case made it clear how far it deviates from this benchmark ideal, and so the risks it raises of undermining the democratic benefits that the ideal seeks to realize.

### Technosolutionism in Public Policy as a Transgression of Spheres

The previous section indicates that technosolutionism can contribute to an undermining of the deliberative democratic dimension of public policymaking. In order to grasp additional negative impacts in the public policy setting, it is helpful to think in terms of different contexts in which technosolutionism can be at work. Technosolutionism originates in the context of technology development at tech companies and start-ups (Morozov, 2013), where values such as creativity, experimentation, speed, playfulness, serendipity and disruption are cherished for their contribution to innovation. In such an environment, starting from an innovative technosolution and trying to think about possible problems that may fit it is not so harmful. However, this same logic *can* become harmful when it is transferred to a different context. As the previous section argued, constructing a problem to fit a predetermined technological solution is harmful *in the context of public policy*. The values important to technology development can indeed be beneficial when the aim is innovation, but they are in some tension with the values and ideals that underpin deliberative democracy, such as careful consideration and reflection, inclusion of a wide range of stakeholders or stakeholder representatives, weighing of diverse perspectives, sequential argumentation, intentionality and focus. It would be strange, in other words, to describe deliberative democracy as embodying in particular the values of creativity, experimentation, speed, playfulness, serendipity and disruption. The exportation of these ideals and values from one context (technological prototyping and product innovation) to a new one (public policymaking for complex societal problems) thus risks crowding out the ideals and values which should be most central in public policymaking.

Moreover, as technosolutionism moves from its original to new contexts, it also brings with it certain actors – namely, those actors who are experts in technosolutions: Big Tech. Elsewhere, we have described this movement of Big Tech actors into new contexts, such as health, education or public policy, and the risks that this movement raises, as “sphere transgressions” (Sharon, 2020, 2021; Sharon & Gellert, 2023; Stevens et al., 2022; Stevens et al., 2024). This can be ascertained from the involvement of Google and Apple in the development of automated contact tracing.

When Apple and Google launched their API for contact tracing, they opted to only allow apps that worked with decentralized storage to build on their API (Apple, n.d.). At that time, discussions about the advantages and disadvantages of centralized versus decentralized contact tracing apps were still ongoing in many countries, with certain countries having already chosen for centralized apps, including Germany, Norway, England and France. With their decentralized protocol, Apple and Google put an abrupt end to this discussion: only apps that worked with decentralized storage would be allowed to run on the API. As we have argued elsewhere, along with other scholars, Google and Apple’s almost complete monopoly on smartphone operating systems meant that the very attempt to automate contact tracing with smartphone applications put public health authorities and governments at the mercy of Google and Apple’s criteria (Lanzing et al., 2022; Veale, 2020; Kuipers, 2020), and they became, in one sweep, ‘global health policy makers’ (Sharon, 2020).

What the focus on technosolutionism highlights is that governments only came to be at the mercy of these tech companies because they were convinced that they needed an app in the first place. That is, because technosolutionism was already at work. In other words, technosolutionism, as a form of decision-making, may precede the transgression of tech actors into the sphere of public policy. The technosolutionist logic allows Big Tech actors to gain power in different societal contexts, in this case inviting them to aid in the containment of COVID-19 and entering the domain of public health. At a time when scholars, governments and civil society are increasingly worried about the growing dependence of public sectors on Big Tech (van Dijck et al., 2018; Taylor, 2021; Lopez Solano et al., 2022), the concept of technosolutionism may thus help us rethink our increasing dependence on Big Tech as a result of the growing influence of technosolutionism, whereby the complex problems society faces are transformed into problems that Big Tech can solve. This, in turn, would suggest that we should be addressing the root cause of this increasing dependence – technosolutionist thinking – rather than only its executors, Big Tech.

### Orphan Problems

A third harm that technosolutionism can cause, based on our research, is that by devoting public resources to solutions for newly constructed problems – arguably, badly-constructed problems – those resources are no longer available for the initial problem that needed solving. The initial problem is thus left without a solution, making it what we call an “orphan problem”. In the case of the Coronamelder, the original problem which motivated the development of the app – the overburdening and undercapacity of the GGD – was forsaken when a new problem – the GGD’s supposed lack of speed and inaccuracy, for which the app *would* be a solution – was defined.^20^ But of course, the original problem never disappeared. We would also argue that the orphan problem in this case *was* a carefully and well-constructed problem. Contact tracing work was the most important virus containment strategy at the time, which had been historically tried and tested. And importantly, the construction of the undercapacity of the GGD as a problem was the result of advice of the OMT^xxx^ and had been adopted by the minister as a precondition for lifting lockdown measures (de Jonge, 2020). Admittedly, a carefully constructed problem may require a bit more. However, by the time the problem construction reached the stage of more democratic scrutiny in parliament, the minister had already proposed the app as a solution, and technosolutionism had kicked in. One could say that the initial problem was still being constructed, but its construction never came to full fruition because technosolutionism was introduced, and “orphaned” it. Even though this problem too presumed a technological solution (a better and more interoperable digital database for the GGD e.g.), this was a tailored technological solution to a simple problem, and the problem construction clearly preceded the solution. It was not a wicked problem, nor a technosolutionist problem, and thereby it was not an example of a technosolution nor a technological fix (see Section 2 for the distinction). In other words, up until that point the orphaned problem did seem to be well- or carefully constructed.

It becomes clear that this carefully constructed problem became an orphan problem when, five months after the first announcement that the government was developing an app, on October 8 2020, the idea to build yet another app, one that would actually support the contact tracing work of the GGDs, reemerged. The idea behind this app, which was developed by the GGDs itself, was to provide the GGDs with information about the contacts of people who tested positive for the coronavirus.^xxxi^ This would alleviate some of the work that the GGD carried out, *supporting* the process of manual contact tracers, and thus addressing the initial problem of undercapacity. The Minister referred to this app as *Solution 2*, and the app would eventually be called *GGD Contact*. It would take another eight months for this app to be launched; nine months after the launch of the Coronamelder.^xxxii^

Despite offering a solution to the initial problem described, the *GGD Contact* app never received the same amount of attention or investment that the Coronamelder did. There may be several reasons for this. First, the *GGD Contact* app may have faced more severe privacy-related problems than the Coronamelder, presenting an important hurdle.^21^ More importantly, one of the underlying causes of the overburdening and undercapacity of the GGD – the original and persistent problem – was partly the result of policy decisions in previous years concerning the organization of the GGD, most notably the broad budget cuts which had led to severely understaffed regional health services (Kuijpers et al., 2020; RVS, 2023; van Heerde, 2020). These structural problems were never issues that a technosolution could have properly addressed. That is to say that addressing the problem of the overburdening of the GGD would have led to difficult questions about how the GGD works and why there is a lack of capacity in the first place, and inevitably to a discussion about the role that cutbacks during the decades preceding the pandemic may have played.

Still, the orphaned problem did persist and even made it back onto the policy table six months later. One way of explaining this delay is that there is only a limited amount of energy, initiative, creativity, and hype that will or can be put into solving a problem. The development of the Coronamelder entailed all of these in great amounts: experts from various areas were called on to contribute to its development; platforms for discussion were created^22^; a largely advertised appathon was organized; there was attention from the national press; and many scholars and engineers were asked to comment.^23^ By the time an initiative to create the second app was set up, one that would try to address the orphaned problem, this energy had dissipated. There was no national press coverage or interest, there were no public calls for experts to contribute.

This creation of orphan problems is another danger of technosolutionism. By redefining a problem so that a specific technology can solve it, attention is focused on a badly and carelessly constructed problem, all the while depleting the resources needed to solve the initial, possibly better constructed, problem. These resources are financial (a whopping 23 million Euros for the Coronamelder (Hulsen, 2021)), but also include things like public attention, discussion, and interest for the orphaned problem, which legitimize the allocation of financial resources towards a solution. This should be a lesson for policymakers engaging in technosolutionist decision-making.

To conclude this section on technosolutionist harms, we want to make an additional point. We contend that our analysis has critical implications for how technology ethics tends to be carried out today: with a predominant focus on the design of the technology, rather than on the broader context, problem definition and actors involved in technology development. The Coronamelder has been praised by many as an ethical success story, upheld as an illustrative example of a technology that takes into account ethical values, in particular values such as privacy and transparency (Martijn, 2023; van der Meulen, 2020). The ethical analysis, commissioned by the Ministry of Health and conducted by a panel of experts, drew up a list of core values which guided its recommendations for the app’s development and implementation (Verbeek et al., 2020). Although these included the recommendation to ‘monitor the social impact of the app’ and its ‘proportionality’^xxxiii^, these recommendations followed after an uncritical acceptance of the app as a solution. While the analysis did discuss conditions for the app to be effective, it did this only by asking: “When is a contact tracing app effective?” Not by asking: “What is the right problem definition?” and “Is a contact tracing app the right solution for that problem?” In short, the ethical analysis did not address nor prevent technosolutionism.

While the Coronamelder did indeed adhere to a list of ethical standards, our analysis shows precisely how it fell short in relation to other and broader democratic values. More specifically, the development of the Coronamelder is an illustration of how ethical reflection – when only applied at the level of design – is a very superficial understanding of ethics: ethics that comes *after* a general solution has already been decided on rather than as a means of arriving at that decision; ethics which only has a say about what a technology will look like, rather than having a say about *if* technology is the best solution in the first place. There is thus a risk that when technosolutionism is at work, ethics, just as problem definitions, begin adapting to technosolutions rather than the other way around. This is not to say that design-focused ethics, such as in the broad fields of “value sensitive design” and “ethics by design” (Friedman, 1997; Hoven et al., 2014; EC, 2021) do not pay attention to what problem is supposed to be solved and how best to frame that problem, but that the inherent focus on design makes them vulnerable to aligning with technosolutionism rather than resisting it. Particular heed to the workings and harms of technosolutionism should thus be paid when these ethical approaches are applied and more thought should be given to *when* ethical intervention occurs, so to also allow for the formulation of non-technological solutions.

## Conclusion

In this paper we advanced a thick conception of technosolutionism which emphasizes the constructivist and backwards-looking aspects of technosolutionism. As opposed to thinner conceptions of technosolutionism which align with the notion of technological fix, this conception allowed us to analyze the Coronamelder not just as a simple techno-fix to a complex problem, but to focus on the various constructions of the problem the Coronamelder was meant to solve. This in turn allowed us to identify how technosolutionism functions in practice and what harms can ensue. We observed how the problem definition of the Coronamelder evolved from being defined as an overburdening and undercapacity of the manual contact tracers of the GGDs, to the assertion that manual contact tracing was not fast and accurate enough. This reformulation of the app’s aim from providing support to being an add-on was not just a semantic slippage but reflected a substantive and consequential transformation of the initial – and we would argue more carefully and better-constructed – problem at hand, reshaping it into something that the Coronamelder could solve.

From our analysis we drew tentative conclusions about how technosolutionist problems are constructed. As a mechanism, we found that this construction, not unsimilar to the construction of scientific facts (Latour & Woolgar, 1979), involves moments of contestation and negotiation before a new problem definition stabilizes, which then seems to have always been there. More emblematic of technosolutionism may be that contestation is not properly addressed, but tends to fade out, and that the problem definition follows tweaks in the technology. As the technology becomes more concrete, its affordances become more apparent, and the technosolutionist problem is constructed to fit these affordances.

We proceeded to identify a number of harms that technosolutionism can provoke. The first is a harm to democracy: by working from a fixed solution towards a problem definition, technosolutionism involves a subversion of an ideal deliberative democratic decision-making process. Technosolutionist problem construction in public policy settings lacks the extensive deliberation, debate and democratic scrutiny which should underpin policy making, ideally if not in practice. In other words, in terms of deliberative democracy, technosolutionist problems are *badly* constructed. Second, as it moves from the sphere of digital innovation to the sphere of public policy, technosolutionism can be seen as a “sphere transgression” (Sharon, 2020, 2021; Sharon & Gellert, 2023; Stevens et al., 2022; Stevens et al., 2024), bringing with it new types of expertise and experts, in this case Google and Apple. In the sphere of public policy this transgression creates dependencies on tech actors for policy solutions. Much like technosolutionist problem construction, this dependency is also constructed: it follows from the taking-for-grantedness that technology is the right solution. We thus argue that in the debate about the growing influence of Big Tech and its threats to democracy, the power of technosolutionism needs to be taken into account. Third, a pernicious by-product of technosolutionism is the creation of what we have called “orphan problems”, whereby the initial problems that triggered the need to develop a (techno)solution are left behind. In the case of the Coronamelder, the initial problem, the overburdening and undercapacity of the GGD, never disappeared, and by the time it became clear that it was still a problem, fewer resources were available to address it.

There are several interesting questions that our analysis raises. Under which conditions does technosolutionism thrive? How does it relate to existing power dynamics? How does it interrelate with a given economic and political climate? In the specific case of the Coronamelder, were all of the actors involved “technosolutionist-oriented”, or were certain parties more favorable, or conversely, more vulnerable to it than others? The scope of our field work and analysis here prevents us from engaging with these questions, which we believe would be interesting questions for future research. This article provides a first step in this direction.

Our contribution is thus multiple. We have advanced a richer concept of technosolutionism, and, through the case of the Coronamelder app, shown what technosolutionism in the context of public policy making does, how to recognize it, and which harms it can cause. Finally, we contend that in order to address these concerns, the focus on technology *design,* which underpins many current technology ethics approaches, is problematic. Such a focus may be *post-factum*, when what is needed is increased ethical intervention *ex-ante,* when it is still possible to ask if technology is the best solution to a given problem at all.

## Endnotes

^i ^Bijlage (blg-929506) Kamerstukken II, 25295 nr. 219 (2020, 7 april): *Advies n.a.v. 63e OMT COVID-19.*

^ii ^Aanhangsel Handelingen II, nr. 67, item 2 (2020, 8 april). p. 37: ‘Hoeveel mensen moeten meedoen om die app nuttig te laten zijn? In de recente, op zichzelf genomen nog wel schaarse, literatuur wordt gesproken over 60% van de bevolking. (…) Want die 60% moeten we dan wel zien te halen. En dat gaan we doen’.

^iii ^Aanhangsel Handelingen II, nr.96, item 6 (2020, 2 september). 

p. 5: ‘Je wil natuurlijk dat de app zo veel mogelijk wordt gebruikt, maar ik ga daar op voorhand geen getal op plakken. Ik zou willen dat er niet meer meldingen in beeld komen dan daadwerkelijk terecht zou zijn, maar ik kan daar nu geen getallen op plakken. Ik denk dat we er gewoon maar mee aan de slag moeten en dat we dan moeten kijken wat de effectiviteit is. Ik denk dat een getalsmatige doelstelling daar op voorhand niet heel erg bij helpt’.

^iv ^Kamerstukken II, 25295 nr. 874 (2021, 13 januari), *Brief van de minister van volksgezondheid, welzijn en sport*. P. 32: ‘Beoogd effect: ‘sneller besmette personen vinden’.

^v^ Bijlage (blg-929506) Kamerstukken II, 25295 nr. 219 (2020, 7 april), Advies n.a.v. 63e OMT COVID-19. p. 4: ‘zo snel mogelijk de mogelijkheden voor ondersteuning van bron- en contactopsporing m.b.v. mobiele applicaties te onderzoeken.’

^vi^ De Jonge, 2020. ‘die apps hebben we eigenlijk nodig om het normale ggd werk te ondersteunen’.

^vii^ Ministerie VWS (2020a). p.1: ‘Slimme digitale oplossingen’.

^viii^ Ministerie VWS (2020a). p.1: ‘om z.s.m. de mogelijkheden voor ondersteuning van bron- en contactopsporing m.b.v. mobiele applicaties te onderzoeken om belasting van de GGD te reduceren’.

^ix^ Aanhangsel Handelingen II, nr. 68, item 2 (2020, 16 april). p. 40: ‘Het gaat om de vraag of het daadwerkelijk het normale werkproces van de GGD ondersteunt. Ondersteunt het daadwerkelijk bron- en contactonderzoek? Dat is het doel en aan dat doel moet het voldoen.’

^x^ Kamerstukken II, 25295, nr. 316 (2020, 21 april). *Verslag van een technische briefing*. p. 5: ‘Bij alles wat gedaan is, heeft die vraag, namelijk het doel van het verkennen van de mogelijkheid van digitale ondersteuning, steeds vooropgestaan’

^xi^ Kamerstukken II, 25295, nr. 351 (2020, 19 mei): *Brief van de minister van volksgezondheid, welzijn en sport*. p. 17: ‘in aanvulling op bestaand bron- en contactonderzoek’

^xii^ Kamerstukken II, 25295, nr. 428 (2020, 24 juni): *Brief van de minister van volksgezondheid, welzijn en sport*. p. 17: ‘dat vult het reguliere bron- en contactonderzoek aan’

^xiii^ Bijlage (blg-929506) Kamerstukken II, 25295, nr. 460 (2020, 3 juli) *Advies 3: relatie notificatie-app en covid-19 teststrategie*. p.2: ‘De toegevoegde waarde van de notificatie-app bovenop het reguliere bron- en contactonderzoek’

^xiv^ Aanhangsel Handelingen II, nr.96, item 6 (2020, 2 september). p. 3: ‘als aanvulling op het bron- en contactonderzoek.’

^xv^ Ministerie VWS (2020c). p. 4: ‘de realisatie en implementatie moeten echter geen nadelig gevolg hebben op de bestaande GGD-processen’

^xvi^ Bijlage (bls-945067) Kamerstukken II, 25295, nr. 460 (2020, 14 juli) *Ethische analyse van de COVID- 19 notificatie-app ter aanvulling op bron en contactonderzoek GGD*. p. 12: ‘Het is van belang om vast te stellen hoeveel werk de app met zich meebrengt voor de GGD: kan het huidige systeem het aan?’

^xvii^ Ministerie VWS (2020b). ‘We zijn op deze zaterdag bij elkaar om een van de grote vraagstukken van onze tijd op te lossen: hoe krijgen we de verspreiding van het coronavirus beter in kaart? Hoe kunnen we de contacten van besmette mensen beter en sneller opsporen? En daarmee de snelle verspreiding van het virus indammen? Niet alleen om levens te redden, ook om ons land van het slot af te krijgen. Om slim uit de intelligente lockdown te komen waar we nu al ruim een maand in zitten.’

^xviii^ Ministerie VWS (2020b). ‘Wat kan een app, wat jullie niet kunnen?’

^xix^ Ministerie VWS (2020b). ‘Je dan niet hoeft te vertrouwen op het geheugen van de patiënt, je contacten kunt traceren waar we ons mogelijk niet van bewustzijn of die we vergeten zijn, en het proces daarmee kan versnellen.’

^xx^ Aanhangsel Handelingen II, nr. 67, item 2 (2020, 8 april). p. 37: ‘Hoeveel mensen moeten meedoen om die app nuttig te laten zijn? In de recente, op zichzelf genomen nog wel schaarse, literatuur wordt gesproken over 60% van de bevolking. (…) Want die 60% moeten we dan wel zien te halen. En dat gaan we doen.’

^xxi^ Aanhangsel Handelingen II, nr.96, item 6 (2020, 2 september). p. 5: ‘Je wil natuurlijk dat de app zo veel mogelijk wordt gebruikt, maar ik ga daar op voorhand geen getal op plakken. Ik zou willen dat er niet meer meldingen in beeld komen dan daadwerkelijk terecht zou zijn, maar ik kan daar nu geen getallen op plakken. Ik denk dat we er gewoon maar mee aan de slag moeten en dat we dan moeten kijken wat de effectiviteit is. Ik denk dat een getalsmatige doelstelling daar op voorhand niet heel erg bij helpt.’

^xxii^ Kamerstukken II, 25295 nr. 874 (2021, 13 januari): *Brief van de minister van volksgezondheid, welzijn en sport*. p. 32: ‘Van de mensen die bij het aanvragen van een test aangaven dat ze een melding van CoronaMelder hadden gehad was tot 27 november 99,8% op het moment van het aanvragen van de test nog niet benaderd door de GGD vanuit het reguliere bron- en contactonderzoek. Dit indiceert dat CoronaMelder tot dat moment inderdaad sneller nauwe contacten bereikt.’

^xxiii^ Aanhangsel Handelingen II, nr. 67, item 2 (2020, 8 april). p. 37: ‘Hoeveel mensen moeten meedoen om die app nuttig te laten zijn? In de recente, op zichzelf genomen nog wel schaarse, literatuur wordt gesproken over 60% van de bevolking. (…) Want die 60% moeten we dan wel zien te halen. En dat gaan we doen.’

^xxiv^ Aanhangsel Handelingen II, nr.96, item 6 (2020, 2 september). p. 5: ‘Je wil natuurlijk dat de app zo veel mogelijk wordt gebruikt, maar ik ga daar op voorhand geen getal op plakken. Ik zou willen dat er niet meer meldingen in beeld komen dan daadwerkelijk terecht zou zijn, maar ik kan daar nu geen getallen op plakken. Ik denk dat we er gewoon maar mee aan de slag moeten en dat we dan moeten kijken wat de effectiviteit is. Ik denk dat een getalsmatige doelstelling daar op voorhand niet heel erg bij helpt.’

^xxv^ Kamerstukken II, 25295 nr. 874 (2021, 13 januari), *Brief van de minister van volksgezondheid, welzijn en sport*. P. 32: ‘Beoogd effect: ‘sneller besmette personen vinden’

^xxvi^ Bijlage (945057) Kamerstukken II, 25295, nr. 460 (2020, juni): *Advies 1: Programma van Eisen voor digitale oplossing ter aanvulling op bron- en contactonderzoek GGD*. P. 2: ‘Het PvE lijkt toe te werken naar een specifieke oplossing, namelijk de creatie van een specifieke notificatie-app, in plaats van een concreet en generiek overzicht te geven van de randvoorwaarden en eisen voor deze vorm van digitale ondersteuning te formuleren.’

^xxvii^ Kamerstukken II, 25295, nr. 428 (2020, 24 juni): *Brief van de minister van volksgezondheid, welzijn en sport*. P. 17: ‘De Begeleidingscommissie adviseert om het doel van de notificatie-app nader te specificeren en om succes- en faalcriteria op te stellen. Het doel staat voor mij voorop. Dat is om die contacten die besmette personen zich niet herinneren snel te laten weten dat ze in contact zijn geweest met iemand die besmet is met het virus. Dat vult het reguliere bron- en contactonderzoek aan en leidt tot meer, en sneller, gewaarschuwde contacten. Elke gevonden extra besmetting is winst, omdat daarmee een mogelijke verspreidingsketen wordt verbroken. Ook bij een lage adoptiegraad is al effect te verwachten.’

^xxviii^ Bijlage (945068) Kamerstukken II, 25295, nr. 460 (2020, 3 juli): *Advies 3: relatie notificatie-app en covid-19 teststrategie*. P. 2: ‘De toegevoegde waarde van de notificatie-app bovenop het reguliere bron- en contactonderzoek is het sneller kunnen opsporen en notificeren van contacten, inclusief anonieme contacten en contacten zonder klachten die wel al besmettelijk zijn en daarmee verdere transmissie te voorkomen.’

^xxix^ Conversation on Slack *#Coronamelder*-channel in workspace *CodeforNL*, on april 19th 2020. User 1: ‘contacten tussen bekende mensen zal het werk niet zijn voor de GGD denk ik. Ik weet wel bij wie ik op bezoek ga (nu betrekkelijk weinig). Het gaat hem om die onbekende in de trein die de GGD nu niet kan benaderen’ […] User 2: ‘maar dat is niet de argumentatie van de GGD waarom ze een app willen hè. Want ze zeggen dat het een schaalbaarheidsprobleem is. Niet een probleem van te weinig data.’

^xxx^ Bijlage (blg-929506) Kamerstukken II, 25295 nr. 219 (2020, 7 april): *Advies n.a.v. 63e OMT COVID-19*.

^xxxi^ Kamerstukken II, 25295, nr. 620 (2020, 8 oktober): *Brief van de minister van volksgezondheid, welzijn en sport*. p. 5: ‘in het kader van digitale ondersteuning van het bron- en contactonderzoek’ (…) ‘Dit stelt mensen die positief zijn getest op het coronavirus onder meer in staat zelf digitaal gegevens over hun contacten aan te leveren bij de GGD. Hierdoor hoeft die inventarisatie niet (in zijn geheel) uitgevoerd te worden door een GGD-medewerker’

^xxxii^ Kamerstukken II, 25295, nr. 1396 (2020, 13 augustus 2021): *Brief van de minister van volksgezondheid, welzijn en sport*.

^xxxiii^ Bijlage (bls-945067) Kamerstukken II, 25295, nr. 460 (2020, 14 juli) *Ethische analyse van de COVID-19 notificatie-app ter aanvulling op bron en contactonderzoek GGD*. p. 2: ‘De overheid dient de sociale impact van de app zorgvuldig te monitoren aan de hand van de in deze beoordeling aangereikte principes.’

## Data Availability

All data used for this study are publicly available. The references for the relevant documents and the original Dutch text on which the study is based can be found in the appendix.

## Footnotes

1 Researcher at the Dutch Rathenau Institute in a debate organized about the introduction of the Dutch contact tracing app and privacy: https://ibestuur.nl/artikel/als-die-app-het-antwoord-is-wat-is-dan-eigenlijk-de-vraag/

2 In this sense, Morozov is not a constructivist at heart, insofar as he allows for a distinction to be upheld between “real” and “constructed” problems, which constructivists would object to. Granted, Morozov is not writing within a tradition of academic constructivism, and his aims and contribution with regards theorizing technsolutionism lie elsewhere.

3 Exceptions we have found are: Pfotenhauer et al.’s (2022) article on the “politics of scaling”, Campbell-Verduyn & Gstrein’s (2024) work on contact tracing, Kuusela and Kantola (2023) in their use of the concept of *Unpolitical Solutionism*.

4 See Table 2 for descriptions of main organizations involved in the development of the Coronamelder.

5 All information and quotes from policy documents are referred to by their document type, number and page number in the appendix, accompanied by the original Dutch quote.

6 All emphases in quotes in this section have been added.

7 These promises were not new in the broader discussion on the implementation of contact tracing apps, but this is the first time they were foregrounded in the discussion on the Dutch contact tracing app that should support the GGD contact tracing.

8 There is a difference between downloaded apps and apps in use. Though the app works in the background, the Bluetooth function needs to be on for the app to be in use. The numbers only show who downloaded the app.

9 To be clear, only people using the app are in this group.

10 We have not, however, been able to carry out an elaborate ethnography of the construction of the problem as Latour and Woolgar do in *Laboratory Life*, which would require observation during the development of the Coronamelder and speaking with problem constructors. We hope that our work can inspire others to undertake more elaborate ethnographic research on the construction of (technosolutionist) problems in public policy.

11
https://codefor.nl

12 For example, one user suggested that the shift toward the new problem – finding strangers on a train – made for ‘pretty PowerPoint presentations’ (Slack conversation April 19 2020).

13 Affordances entail looking at ‘the possibilities for action that it offers’ (Hutchby, 2001). Technology, in this understanding, makes possible certain actions, just as it prohibits others. In our case, these actions the technology makes possible are the solving of some problems, while prohibiting the solving of other problems.

14 In his later work *An Inquiry into Modes of Existence* (2013), looking back at the heated discussions around constructivism and the tendency to equate it with relativism, Latour casts doubt on the usefulness of the term, fearing that it will always imply that constructed things are “false” as opposed to “real” or “objective” (p. 154). He proposes to use the term “instauration” instead (p. 160). Here, we stick to the term constructivism, as we specifically invoke the discussion on constructivism of science to highlight the similarities and differences between the construction of scientific facts and the construction of problems. The use of “instauration” would not enable us to connect to this discussion in the same way.

15 The DPG’s, Directeuren Publieke Gezondheid, who are responsible for the GGD and GHOR.

16 For an elaborate analysis of this appathon’s democratic potential and deficits see Ozkaramanli et al. (2022)

17 This is not to say that there is anything inherent to either the use of technology to solve a given problem, or an appathon for testing technological solutions, which *necessarily* pushes decision-making into the technosolutionist form. An appathon might be part of a broader democratic decision-making process, for example, when it is organized after the problem and solution definitions have first been properly debated, in order to test different versions of the solution. However, when technologies are proposed as solutions and when hackathons are part of public policy processes, this should raise a red flag that possibly technosolutionism is at work and is subverting an ideal form of democratic decision-making.

18 The many experts the government had asked to join in the appathon told the Ministry that these apps did not meet their standards. Most importantly, the Dutch Personal Data Protection Authority (Autoriteit Persoonsgegevens) could not condone any of the apps, because there was too much unclarity about its aim. As long as the aim was unclear, the AP could not judge whether the deployment of the app was proportional (Autoriteit Persoonsgegevens, 2020).

19 We thank Henk Wagenaar for drawing our attention to this.

20 Besides our analysis in Section 3, this is shown by the reaction of the GGDs to the app during development, with some GGDs not standing behind the development (see Onderzoeksraad voor Veiligheid, 2022, p. 128), as well as disappointment with the app after development, specifically with the effectiveness of the app for contact tracing work (see Onderzoeksraad voor Veiligheid, 2023, p. 82).

21 Manual contact tracing work done by the GGD involved the handling of a lot of personal data, contact details of possibly infected persons as well as the obvious health data of infection. Though the GGD already handles these types of data, digitizing this work would involve new privacy risks, since these data may have to be stored in a more central place and be more accessible in order to use it for the contact tracing work.

22 Such as the Slack channel ‘Code for NL’, see section 4.3 for further elaboration.

23 See for example the ethical analysis (Verbeek, 2020; Verbeek et al., 2020), scientific discussion meetings (Gezondheidsraad, 2020) or the roundtable discussions with experts by Parliament (https://www.tweedekamer.nl/debat_en_vergadering/commissievergaderingen/details?id=2020A01700), among others.
